# Dual role of pectin methyl esterase activity in the regulation of plant cell wall biophysical properties

**DOI:** 10.3389/fpls.2025.1612366

**Published:** 2025-07-04

**Authors:** Marçal Gallemí, Juan Carlos Montesinos, Nikola Zarevski, Jan Pribyl, Petr Skládal, Edouard Hannezo, Eva Benková

**Affiliations:** ^1^ Develomental Biology Department, Institute of Science and Technology Austria (ISTA), Klosterneuburg, Austria; ^2^ Central European Institute of Technology (CEITEC-MU), Masaryk University, Brno, Czechia

**Keywords:** AFM, hypocotyl, auxin, brassinosteroid, *Arabidopsis thaliana*, cell elongation, cell wall, pectin

## Abstract

**Introduction:**

Acid-growth theory has been postulated in the 70s to explain the rapid elongation of plant cells in response to the hormone auxin. More recently, it has been demonstrated that activation of the proton ATPs pump (H^+^-ATPs) promoting acidification of the apoplast is the principal mechanism by which auxin and other hormones such as brassinosteroids (BR) induce cell elongation. Despite these advances, the impact of this acidification on the mechanical properties of the cell wall remained largely unexplored.

**Methods:**

Here, we use elongation assays of *Arabidopsis thaliana* hypocotyls and Atomic Force Microscopy (AFM) to correlate hormone-induced tissue elongation and local changes in cell wall mechanical properties. Furthermore, employing transgenic lines over-expressing Pectin Methyl Esterase (PME), along with calcium chelators, we investigate the effect of pectin modification in hormone-driven cell elongation.

**Results:**

We demonstrate that acidification of apoplast is necessary and sufficient to induce cell elongation through promoting cell wall softening. Moreover, we show that enhanced PME activity can induce both cell wall softening or stiffening in extracellular calcium dependent-manner and that tight control of PME activity is required for proper hypocotyl elongation.

**Discussion:**

Our results confirm a dual role of PME in plant cell elongation. However, further investigation is needed to assess the status of pectin following short- or long-term PME treatments in order to determine if pectin methyl-esterification might promote its degradation as well as the role of PME inhibitors upon PME induction.

## Introduction

Plants, as sessile organisms, developed exceptional plasticity of growth and development to adapt to their ever-changing environment. One of the most prominent mechanisms to ensure adjustability is rapid elongation growth that allows plant organs to reach a position optimal for utilization of light, nutrients, and survival. Plant cells grow mainly by anisotropic expansion, which results from the interplay between turgor pressure and a fine-tuned local cell wall (CW) relaxation (Olivier et al., 2022; Wolf et al., 2012; Braidwood et al., 2014).

Plant hormone auxin is a principal endogenous regulator of elongation growth, which has been shown to induce cell expansion in plant organs such as stems, coleoptiles, or hypocotyl segments within minutes (Rayle and Cleland, 1992; Fendrych et al., 2016). The current model of auxin-regulated cell expansion is based on the acid growth theory (reviewed in Mockaitis and Estelle, 2008; Perrot-rechenmann, 2010). In this model, auxin induces the transcription of *SAUR* (*Small Auxin Upregulated*) genes, which indirectly activate the proton-pumping plasma membrane (PM)-H^+^-ATPase, thereby enhancing the transport of protons into the extracellular space. Recently, it has been shown that auxin can regulate PM-H^+^-ATPase through yet another mechanism mediated by receptor-like transmembrane kinase1 (TMK1) (Lin et al., 2021). At low apoplastic pH, the activity of CW remodeling proteins such as expansins is enhanced, which leads to CW relaxation through a mechanism that is still not clear (Cosgrove, 2018). In parallel to CW acidification, auxin in the nuclei enhances the transcription of genes including those encoding PM-ATPase, K^+^ channels, expansins, and other CW remodeling enzymes (Majda and Robert, 2018; Velasquez et al., 2016). Similarly to auxin, another class of plant hormones, brassinosteroids, has been reported to induce hypocotyl elongation through the activation of PM-H^+^-ATPase and regulate the expression of several CW remodeling enzymes (Caesar et al., 2011; Minami et al., 2019).

Although many components such as cellulose are important for CW structure, pectins are considered to be principal determinants of the regulation of its bio-physical properties (Saffer, 2018; Shin et al., 2021). They are polymers composed of α-d-galacturonic acid (Gal-A) forming homogalacturonan (HG) linear chains. Pectins are, by default, transported in a methyl-esterified state to the apoplast, where they can be de-esterified by pectin methyl-esterase (PME) proteins in a process that liberates protons. The activity of PMEs is fine-tuned by pectin methyl-esterase inhibitors (PMEIs). *In vitro* assays showed that at the low-pH interaction of PMEIs with PMEs is stabilized and the PME activity is attenuated (Ludivine et al., 2017). De-methylesterified pectins have free carboxyl groups that are hypothesized to either form cross-links mediated by calcium ions (the so-called egg-box hypothesis), which might increase the rigidity of the CW (Hocq et al., 2017), or undergo cleavage to shorter HG chains resulting in the softening of the CW (Cosgrove, 2022). Based on super-resolution microscopy, it has been described that pectins possibly form nanofilaments that alter their quaternary structure upon de-methylesterification and calcium cross-linking, supporting the calcium cross-linking model (Haas et al., 2020). Recently, the egg-box model has been revised to the zipper model, defining that calcium cross-links occur just with free carboxyl sites and not involving hydroxyl interaction (Irabonosi et al., 2025).

Despite the importance of PMEs and PMEIs in the modulation of the pectin structure, there is still lack of mechanistic understanding of their functions in the regulation of cell growth processes, and contradictory results have been published on the effects of those proteins (Jonsson et al., 2022; Cosgrove, 2022). While some studies have reported increased CW stiffness in epidermal cells of hypocotyls overexpressing *PME5* (Bou Daher et al., 2018), others have shown that enhanced PME5 activity results in decreased stiffness of the CW (Peaucelle et al., 2011, 2015). Although both studies were done in dark-grown hypocotyls and scanning close to the base cells, other experimental differences like time of scan [19 h post-germination (HPG) in Peaucelle et al. (2015) and 48 HPG in Bou Daher et al. (2018)] might explain the obtained differences. Lines overexpressing *PMEIs* seem to raise controversy as well. Overexpression of *AtPMEI3* has been associated with increased stiffness of hypocotyl epidermal CWs, but softening effects on CWs have also been reported, both correlating with reduced cell length and growth anisotropy (Bou Daher et al., 2018; Peaucelle et al., 2008, 2011, 2015). *Arabidopsis* plants with increased levels of *AtPMEI2* show enhanced growth and longer roots as a result of promoted cell elongation (Lionetti et al., 2007, 2010; Liu et al., 2018). The expression of *AtPME1* in tobacco pollen tubes inhibited elongation, whereas *AtPMEI2* expression led to an increase in their elongation rate (Röckel et al., 2008). Thus, it seems that a developmental or cellular context needs to be taken into account to explain the role of PME and PMEI in growth processes, their impact on CW mechanical properties, and cell elongation.

Atomic force microscopy (AFM) techniques have recently aroused as an important tool to monitor the bio-physical properties of plant CW. Here we applied AFM to investigate how selected plant hormones, known for their principal role in the regulation of cell elongation, affect CW mechanical properties. We further applied this technique to address a question about how modulation in PME activity affects CW mechanics in relation to calcium levels in the apoplast. We demonstrate a dual role of PME activity, indicating that a narrow window of pectin methylesterification is important for proper cell elongation.

## Results and discussion

### Apoplastic acidification is necessary and sufficient to induce cell elongation through CW stiffness decrease

Modulation of hypocotyl elongation growth is one of the prominent adaptive responses to changes in environmental conditions such as temperature or light intensity. At the molecular level, hypocotyl growth is governed by auxin, which, in concert with other hormonal pathways including brassinosteroids (BR) or gibberellins (GA), promotes cell expansion. Acidification of the apoplast triggered by auxin is one of the essential regulatory steps in this process (Oh et al., 2014). While the promoting effect of acidification on hypocotyl expansion is firmly established, its impact on CW bio-physical properties is still poorly understood. Old reports using extensometers had shown that stems are able to stretch more after apoplastic acidification (Cosgrove, 1993). However, the latest results point out that tensile stretching might not correlate with CW mechanical properties quantified by other techniques like indentation stiffness or apparent Young’s modulus (*E_a_*) obtained from AFM (Zhang et al., 2019).

To establish experimental conditions to investigate the impact of apoplastic acidification on cell elongation and CW bio-physical properties, we adopted an assay using hypocotyl segments of etiolated *Arabidopsis* seedlings (Schenck et al., 2010; Fendrych et al., 2016). Initially, employing the ApopH apoplastic pH sensor (Gjetting et al., 2012), we confirmed that incubation of etiolated hypocotyl segments of 3-day-old seedlings in either acidic (pH 3.5) or alkaline (pH 9.0) buffer for 2 h (h) resulted in expected changes of the apoplast pH in the hypocotyl epidermis (**Supplementary Figures S1A, B**). Comparably to the incubation in acidic buffer, treatment with 10 μM synthetic auxin 1-naphthylacetic acid (NAA) at pH 6.0 for 2 h led to acidification of the apoplast (Fendrych et al., 2016), while auxin was not able to trigger acidification of the apoplast when a buffer of pH 9.0 was used (**Supplementary Figures S1A, B**).

**Figure 1:**
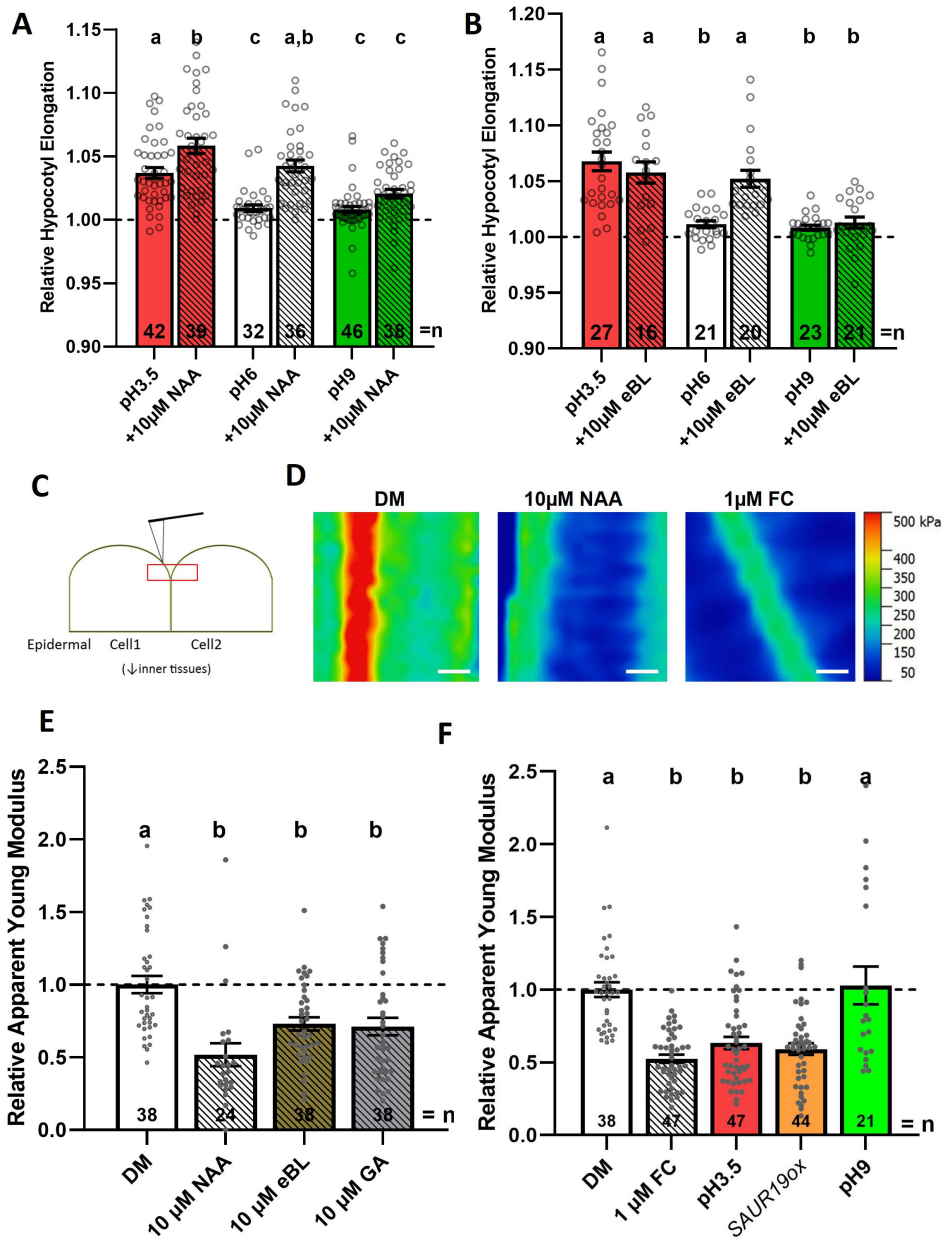
Acidification induces hypocotyl segment elongation and CW softening. **(A, B)** Relative hypocotyl segment length after 3 h of the indicated treatments. Hypocotyl segments of 3-day-old seedlings were incubated in depletion medium (DM) adjusted to pH 3.5, 6.0, or 9.0 with or without auxin (10 µM 1-naphthaleneacetic acid, NAA) **(A)** and BR (10 µM epibrassinolide, eBL) **(B)**. Dashed lines indicate the relative initial length of hypocotyl segments. **(C)** Scheme of the experimental setup used for AFM measurements. Periclinal cell walls, parallel to growth axis, between two hypocotyl epidermal cells (indicated by the red square), were scanned using a 25 × 25-µm area. **(D)** Representative AFM scans in a heat map color code of periclinal epidermal CWs for hypocotyl segments of 3 day-old seedlings incubated for 2 h in depletion medium (DM) adjusted to pH 6.0 supplemented with auxin (10 µM NAA) and FC (1 µM). Scale bar = 5 µm. **(E, F)** Relative apparent Young’s modulus (*E_a_*) of selected CW regions of 3-day-old seedlings incubated for 2 h in depletion medium (DM) adjusted to pH 6.0 and supplemented with auxin (10 µM NAA), brassinosteroid (10 µM eBL), and gibberellin (10 µM GA) **(E)** and in DM adjusted to pH 3.5 and pH 9.0, supplemented with fusicoccin (1 µM FC in DM, pH 6.0) and SAUR19ox (in DM, pH 6.0) **(F)**. Dashed lines indicate normalized *E_a_* with respect to DM. Bars represent average ± SE, and dots represent individual data points. *N* indicates the total number of hypocotyl segments quantified **(A, B)** and the total number of scan maps performed and analyzed **(C, D)**. Tukey–Kramer test was performed, and significant differences are shown as the letters above each bar.

Measurements of hypocotyl length showed that 3 h of incubation in the acidic buffer was sufficient to trigger elongation in a similar range as hormone auxin or BR (epibrassinolide, eBL) at pH 6.0 [with elongation rates that are very similar to those previously published by Fendrych et al. (2016)]. Alkaline buffer did not promote hypocotyl elongation and interfered with hormone-induced elongation (**Figures 1A, B**). Importantly, an analysis of the auxin-sensitive reporter R2D2 (Liao et al., 2015) showed that auxin response is not affected by the alkaline pH itself (**Supplementary Figures S1C, D**). Intriguingly, auxin is still able to trigger more elongation than just acidification (comparing pH 3.5 vs. pH 3.5 + NAA-treated hypocotyls). This might be due to other auxin signaling effects not related to pH changes in the apoplast, as CW synthesis, like new cellulose deposition or activation of other CW remodeling enzymes.

Thus, consistently with previous findings, our experimental setup using *Arabidopsis* hypocotyls shows that the acidification of the apoplast, achieved either through incubation in acidic buffer or hormonal treatments, is both sufficient and necessary for hypocotyl cell elongation (Cleland et al., 1991; Rayle and Cleland, 1992; Arsuffi and Braybrook, 2018).

To establish and validate our AFM experimental platform for measurements of CW mechanical properties, we inspected how the CW rigidity of hypocotyl epidermal cells is altered in different growth regimes (scanning region shown in **Figure 1C** and representative acquired scan results shown in **Supplementary Figure S2A**). Hence, we measured *E_a_* in either long etiolated or short de-etiolated hypocotyls of wild type (Col-0) or hypocotyls of light-insensitive mutants in phytochrome A and B (*phyAphyB*), which elongate despite growing in light conditions (**Supplementary Figure S2B**). We observed a strong correlation between CW mechanical properties and hypocotyl elongation. The average apparent *E_a_* measured on periclinal CWs (parallel with hypocotyl growth axis) in etiolated hypocotyls was around 441 ± 87 kPa standard error (SE). In light-grown hypocotyls, *E_a_* increased to 3,699 ± 670 kPa, while in the *phyAphyB* mutant (with intermediate hypocotyl elongation) intermediate *E_a_* values of around 1,544 ± 130 kPa were detected (**Supplementary Figures S2C, D**). Those results are largely in agreement with previously published reports using AFM (Peaucelle et al., 2015) and also measurements of CW stiffness using Brillouin scattering microscopy (Elsayad et al., 2016), confirming that our indentation *E_a_* is representative of the CW rigidity.

Applying the same AFM setup, we analyzed the impact of hormonal treatments on the mechanical properties of CWs in hypocotyl segments. Measurements of cell growth by confocal life imaging on those hypocotyl segments confirmed that epidermal cells in the middle part of hypocotyl segments of 3-day-old seedlings rapidly elongate in response to auxin when compared to untreated control (**Supplementary Figure S3**). Thus, analyses by AFM were standardly performed on those epidermal cells in the middle zone of hypocotyl segments.

All treatments, which enhanced hypocotyl elongation including auxin, BR, and GA, decreased CW stiffness at a similar range (60% of *E_a_* compared to the non-treated hypocotyls; 0.52 ± 0.08 SE for auxin, 0.72 ± 0.05 for BR, and 0.71 ± 0.06 for GA; **Figures 1D–F**). As hormones such as auxin and BR can induce apoplastic acidification (Fendrych et al., 2016; Minami et al., 2019), we inspected whether direct acidification of apoplast would induce similar changes in CW stiffness. Apoplastic acidification as a result of incubation of hypocotyl segments in acidic buffer (pH 3.5) for 2 h or the PM-H^+^-ATPase pump activation by either fusicoccin drug (FC, Baunsgaard et al., 1998; Ballio et al., 1964) or overexpression of *SAUR19ox* (Spartz et al., 2012) led to the significant reduction of *E_a_* when compared to wild-type control incubated in a buffer of pH 6.0 (0.67 ± 0.06 for pH 3.5 buffer, 0.53 ± 0.03 for FC, and 0.59 ± 0.04 for *SAUR19ox*). The incubation in an alkaline buffer had no significant effect on the *E_a_* of CW in hypocotyl epidermal cells (**Figures 1D, F**). Hence, we hypothesize that various hormonal treatments, which induce hypocotyl elongation, converge on the regulation of mechanisms that control the acidification of the apoplast and subsequently promote CW softening.

### Auxin-induced softening of the CW requires intact signaling pathway

**Figure 2:**
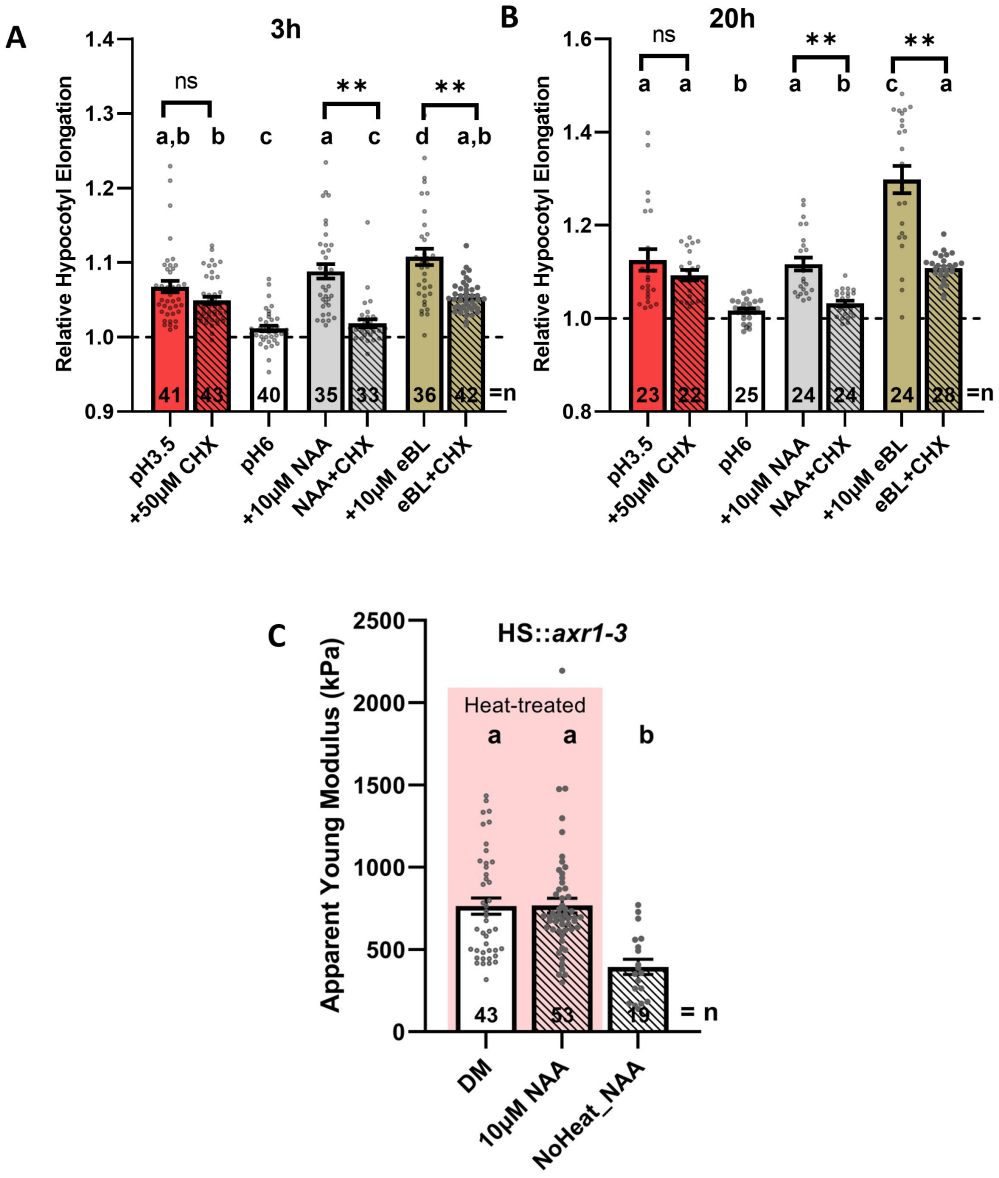
Auxin-triggered hypocotyl elongation is proteosynthesis dependent. **(A, B)** Relative hypocotyl segment length after 3 h **(A)** or 20 h **(B)** of the indicated treatments. Hypocotyl segments of-3 day-old seedlings were incubated in DM medium adjusted to pH 6.0 or 3.5 (red bars) and supplemented with hormones (NAA and eBL) with or without the protein synthesis inhibitor cycloheximide (50 µM CHX), respectively, as indicated. Dashed lines mark the initial relative length of hypocotyl segments. **(C)** Average *E_a_* quantification of periclinal epidermal CWs of 3-day-old hypocotyl segments of *HS::axr3–1* seedlings, not treated or heat-treated for 40 min at 37°C, respectively, and incubated in DM with or without auxin for 2 h. Bars represent average ± SE, and dots represent individual data points. *N* indicates the total number of hypocotyl segments quantified **(A, B)** or scans performed and analyzed **(C)**. Tukey–Kramer test was performed, and significant differences are shown as the letters above each bar. Direct pair significant differences are indicated as **P < 0.01 (*t*-test). ns remaks Not Significant differences.

Our results indicated that hypocotyl elongation triggered by hormones like auxin or BR or apoplast acidification correlates with CW softening. Published works have shown that an intact signaling pathway is required for auxin-induced acidification and hypocotyl elongation (Fendrych et al., 2016) but that BR is able to activate PM-H^+^-ATPs directly (Caesar et al., 2011; Minami et al., 2019). Using cycloheximide (CHX), an inhibitor of proteosynthesis (Rose, 1974), in agreement with previous studies, we observed that auxin-mediated hypocotyl elongation requires protein biosynthesis (Bates and Cleland, 1979; Rayle and Cleland, 1980; Kutschera and Schopfer, 1985; Fendrych et al., 2016), whereas hypocotyl elongation triggered by incubation in acidic buffer (pH 3.5) is not dependent on *de novo* protein synthesis (**Figures 2A, B**). Interestingly, the BR promoting effect on hypocotyl elongation is only partially independent of *de novo* protein synthesis. Hypocotyls elongate more if proteosynthesis is not compromised, suggesting that, in addition to direct activation of the proton pump, another protein-biosynthesis-dependent mechanism might contribute to the regulation of hypocotyl elongation (**Figures 2A, B**). Next, we examined how inhibition of the auxin signaling pathway affects the CW mechanical properties and sensitivity to auxin. Using a well-described heat-inducible *HS::axr 3–1* transgenic line (Knox et al., 2003), we found that accumulation of *axr3-1*, a dominant negative repressor of auxin signaling and auxin-induced hypocotyl elongation, interferes with auxin-mediated softening of CWs when compared to non-heat-treated control (**Figure 2C**), with results that are largely in agreement with previous reports (Fendrych et al., 2016). It is worthy to mention that the induction of the *axr3–1* by heat shock was done previous to the auxin treatment (which was done at room temperature). We conclude that auxin-induced hypocotyl elongation and CW softening are dependent on new protein biosynthesis and intact transduction cascade.

### Balanced PME activity mediates fast hypocotyl elongation

We showed that hormone-induced apoplastic acidification is necessary for fast hypocotyl elongation and CW softening. However, processes associated with the re-structuring of CW that result in modulation of its biophysical properties are still poorly understood. Pectins, as important components of the primary CW, have been proposed as a principal determinant of the mechanical properties of the CW (Shin et al., 2021; Saffer, 2018). In particular, the degree and pattern of methylesterification of homogalacturonan chains controlled by PME and their esteric inhibitors PMEI might have a decisive impact on CW mechanics (Hocq et al., 2017).

To study early responses to transient increase of PME activity, we generated inducible *PME* overexpressing (*ox*) lines. *PME1* as an example of the auxin-inducible homolog of the *PME* family (Steffen et al., 2005; Nemhauser et al., 2006; Simonini et al., 2017) and a previously studied *PME5* were selected for detailed analyses (Peaucelle et al., 2015; Bou Daher et al., 2018). Several independent lines were obtained and selected based on the expression levels determined by RT-qPCR and Western blot (**Supplementary Figures S4A, B**). The expression of *PME1ox* as well as *PME5ox* in seedlings exposed to β-estradiol (β-EST) for 48 h affected the seedling development and resulted in shorter roots compared to non-induced seedlings (**Supplementary Figure S5A**), indicating that recombinant PME1-HA and PME5-HA proteins maintain their activities. Moreover, 24-h induction of *PME*1ox (line #1) triggered higher levels of LM19 antibody binding to CW, confirming a higher degree of methyl-esterified pectin as expected (**Supplementary Figure S5B**).

**Figure 3:**
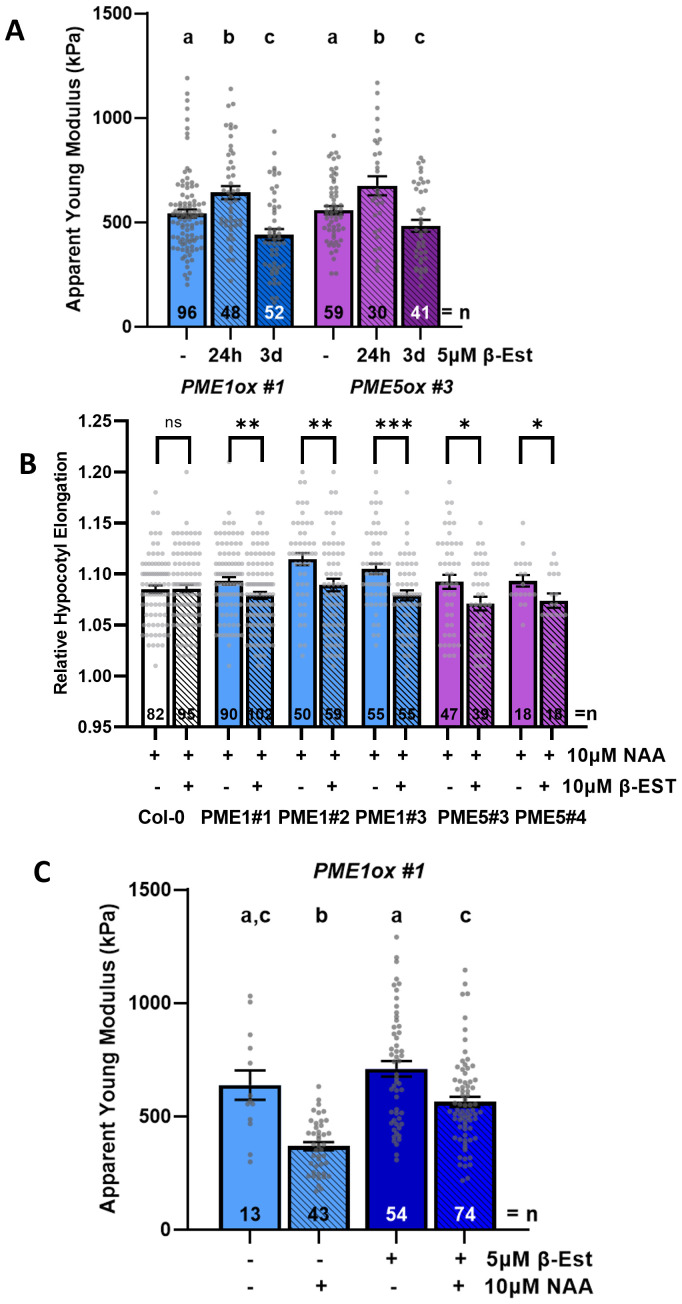
Enhanced expression of *pectin methyl-esterase1* (*PME1*) or *PME5* modulates CW stiffness and interferes with auxin-promoted hypocotyl elongation. **(A)** Average *E_a_* quantification of periclinal CWs measured in the hypocotyl epidermal cells of 3-day-old seedlings of *PMEox*s lines at the indicated induction time with β-estradiol (β-Est). **(B)** Relative hypocotyl segment length in wild-type Col-0 and *PME1ox* and *PME5ox* lines not treated or treated with β-Est for 24 h and auxin (1-naphthaleneacetic acid, NAA) for 3 h as indicated. **(C)** Average *E_a_* quantification of periclinal CWs of hypocotyl epidermis in 3-day-old *PME1ox* seedlings not treated or treated with β-EST for 24 h and hypocotyl segments not treated or treated with auxin (NAA) for 2 h. Bars represent average ± SE, and dots represent individual data points. *N* indicates the total number of hypocotyl segments quantified **(B)** or scans performed and analyzed **(A, C)**. Tukey–Kramer test was performed, and significant differences are shown as the letters above each bar. Direct pair significant differences are indicated as *P < 0.05, **P < 0.01, and ***P < 0.001 (*t*-test).

To examine how the enhanced expression of *PMEs* affects CW mechanical properties, we employed AFM. Monitoring of CW stiffness in hypocotyl epidermal cells revealed surprising, time-dependent effects of PMEs on the CW mechanics. Induction of *PMEs* expression for a short time (~24 h) resulted in stiffer CWs, while *PMEs* expression persisting for about 3 days correlated with CW softening when compared to the untreated control (**Figure 3A**). The enhanced expression of *PME5* has been reported to result in stiffening of CWs in hypocotyl epidermal cells (Bou Daher et al., 2018), while in another study softening of hypocotyl epidermal cells and shoot apical meristem has been detected (Peaucelle et al., 2011, 2015). In light of our observations, we hypothesize that the previously observed softening of CWs might be a result of prolonged and/or very strong PME activity. As specified by the authors (Peaucelle et al., 2015, **Supplementary information**), just seedlings with a high expression and showing strong phenotype defects were selected to be analyzed. In our experiments, we did not apply any pre-selection based on phenotype to choose seedlings for any analysis, and therefore higher variability and lower average expression levels might be expected.

Next, we analyzed how the enhanced expression of *PME1* or *PME5* affects auxin-induced elongation. Intriguingly, growth response to auxin was hampered in hypocotyls in which *PMEs* expression was induced (24 h of induction by β-EST and 3 h of treatment with auxin) when compared to non-induced controls treated with auxin only, indicating that modulation of pectin methylesterification by PMEs interferes with auxin-triggered elongation of hypocotyls (**Figure 3B**). As an enhanced *PME* expression interfered with auxin-induced growth, we tested whether this PME effect correlates with alteration of CW mechanical properties. AFM measurements revealed that *PME* expression significantly attenuates auxin capacity to relax CWs (**Figure 3C**).

These results altogether suggest that tightly controlled pectin de-methylesterification is required for fast hypocotyl elongation in response to auxin and that modulation of the *PME* expression might lead to either softening or stiffening of CW, depending on the duration of the PME activity.

### Calcium availability affects the PME-mediated CW stiffening

Recently, the paradox of how PME activity might lead to two distinct effects on CW mechanics has been discussed, and a model was proposed, in which a pattern of pectin de-methylesterification is an important determiner of CW stiffness (Hocq et al., 2017; Ludivine et al., 2017; Arsuffi and Braybrook, 2018). Blockwise HG de-methylation might lead to the formation of stretches of negatively charged HG chains that can interact with divalent Ca^2+^ ions and form the so-called “zipper” structures, increasing the stiffness of CWs. On the other hand, random de-methylesterification might enhance the cleavage of HG chains by pectin-degrading enzymes to oligogaracturonides and thereby promote CW softening.

**Figure 4:**
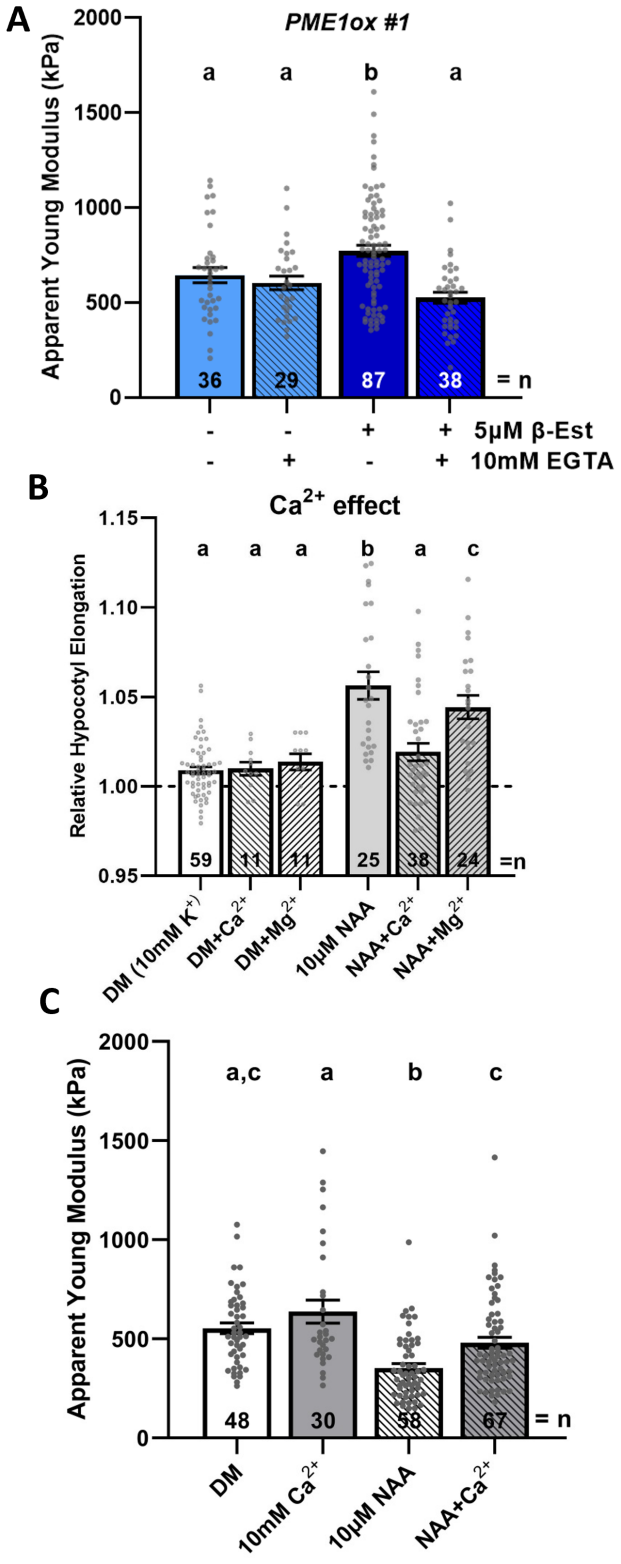
Calcium interferes with auxin-triggered hypocotyl elongation and CW softening. **(A)** Average *E_a_* quantification of periclinal CWs of hypocotyl epidermis in 3-day-old hypocotyl segments from *PME1ox* line not treated or treated for 24 h with β-EST and incubated or not for 2 h in medium supplemented with calcium chelator (ethyleneglycoltetraacetic acid, EGTA). **(B)** Relative hypocotyl segment length after 3 h of the indicated treatments. DM was modified by adding 5 mM CaCl_2_ or MgCl_2_ (together with 5 mM KCl) as ion-interfering treatments. **(C)** Average *E_a_* quantified on periclinal epidermal CWs of 3-day-old hypocotyl segments incubated for 2 h in depletion medium supplemented with auxin (1-naphthaleneacetic acid, NAA) and Ca^2+^ as indicated. Dashed lines indicate the relative initial length of hypocotyl segments. Bars represent average ± SE, and dots represent individual data points. *N* indicates the total number of scans performed and analyzed **(A, B)** and the hypocotyl segments quantified **(C)**. Tukey–Kramer test was performed, and significant differences are shown as the letters above each bar.

Our AFM measurements in hypocotyls pointed out that CW biophysical properties can be modified differently depending on the duration of PME activity (**Figure 3A**). As Ca^2+^ ions appear to be a critical factor determining the rigidity of CWs, we examined whether modulation of Ca^2+^ levels affects CW mechanical properties and the hypocotyl capacity to elongate. To explore whether increased CW stiffness as a result of enhanced *PME1* expression might involve Ca^2+^-mediated cross-linking of HGs, we incubated hypocotyls of *PME1ox* line in the medium supplemented with calcium chelator ethyleneglycoltetraacetic acid (EGTA) (Feng et al., 2018). No changes in CW stiffness were detected in hypocotyl epidermal cells of non-induced *PME1ox* incubated in the medium supplemented with EGTA when compared to non-EGTA-treated controls. The CW stiffness in hypocotyls overexpressing *PME1ox* and treated with EGTA was comparable to that detected in control non-induced hypocotyls (**Figure 4A**). These results suggest that the availability of Ca^2+^ might be critical for cross-linking of de-methylesterified HGs shortly after the activity of *PME* is increased.

Using maize coleoptiles, it has been shown that Ca^2+^ added to the medium interferes with auxin-induced elongation (Tode and Lüthen, 2001). Consistently, we observed that Ca^2+^ added into the depletion medium attenuated the auxin-triggered elongation of *Arabidopsis* hypocotyl segments. When compared to Ca^2+^, divalent Mg^2+^ ions interfered with auxin-induced elongation significantly less (**Figure 4B**). However, whether Ca^2+^ inhibition of auxin-induced hypocotyl elongation involves the alteration of CW mechanical properties has not been investigated. AFM measurements of hypocotyl segments revealed significantly increased CW stiffness in epidermal cells of hypocotyls incubated in the medium supplemented with auxin in the presence of Ca^2+^ when compared to control hypocotyls exposed to auxin only (**Figure 4C**), suggesting that Ca^2+^ availability might significantly affect auxin-mediated softening of CWs.

Our data altogether show that the levels of extracellular calcium play a decisive role in auxin-mediated softening of the CWs and hypocotyl elongation. Furthermore, they support a model in which a short-term PME activity generates a de-methylation pattern of HGs, which is prone to cross-linking by Ca^2+^ that promotes stiffening of CWs.

## Conclusions

The relation between CW composition and its mechanical properties is still a big unsolved enigma. CW remodeling is a dynamic process that is an integral part of regulatory mechanisms controlling the growth and development of plant organs. Modulation of CW biophysical properties is a result of a complex regulatory network encompassing multiple plant hormones, CW remodeling enzymes, calcium ions, and pH (Wolf et al., 2012; Hocq et al., 2017).

Here we show that acidification is a necessary step for CW softening in hypocotyl cells. When acidification by hormones is compromised by an alkaline environment, softening of CWs and growth is significantly attenuated, indicating that it is a shared mechanism among several plant hormones that promote hypocotyl elongation.

Pectin is an important component of CW. A recent report using 3D-STORM microscopy applied on cotyledon pavement cell suggested that the degree of methylation of HGs might determine the quaternary structure that defines intrinsic pectin swelling properties. These observations were summarized in the “expanding beam model” proposing that local HG de-methylesterification leads to pectin nanofilament swelling (Haas et al., 2020, 2021). The authors showed that de-methylesterification of HG alone is sufficient to induce expansion in cotyledon pavement cells. In such context, the balanced activities of PME and PMEI enzymes should be key factors regulating cell growth. Accordingly, previous reports have demonstrated that PME and PMEI control the CW biophysical properties (Bou Daher et al., 2018; Peaucelle et al., 2015; Jonsson et al., 2021), suggesting that de-methylesterification of HGs is an essential determiner of cell expansion and tissue growth.

**Figure 5:**
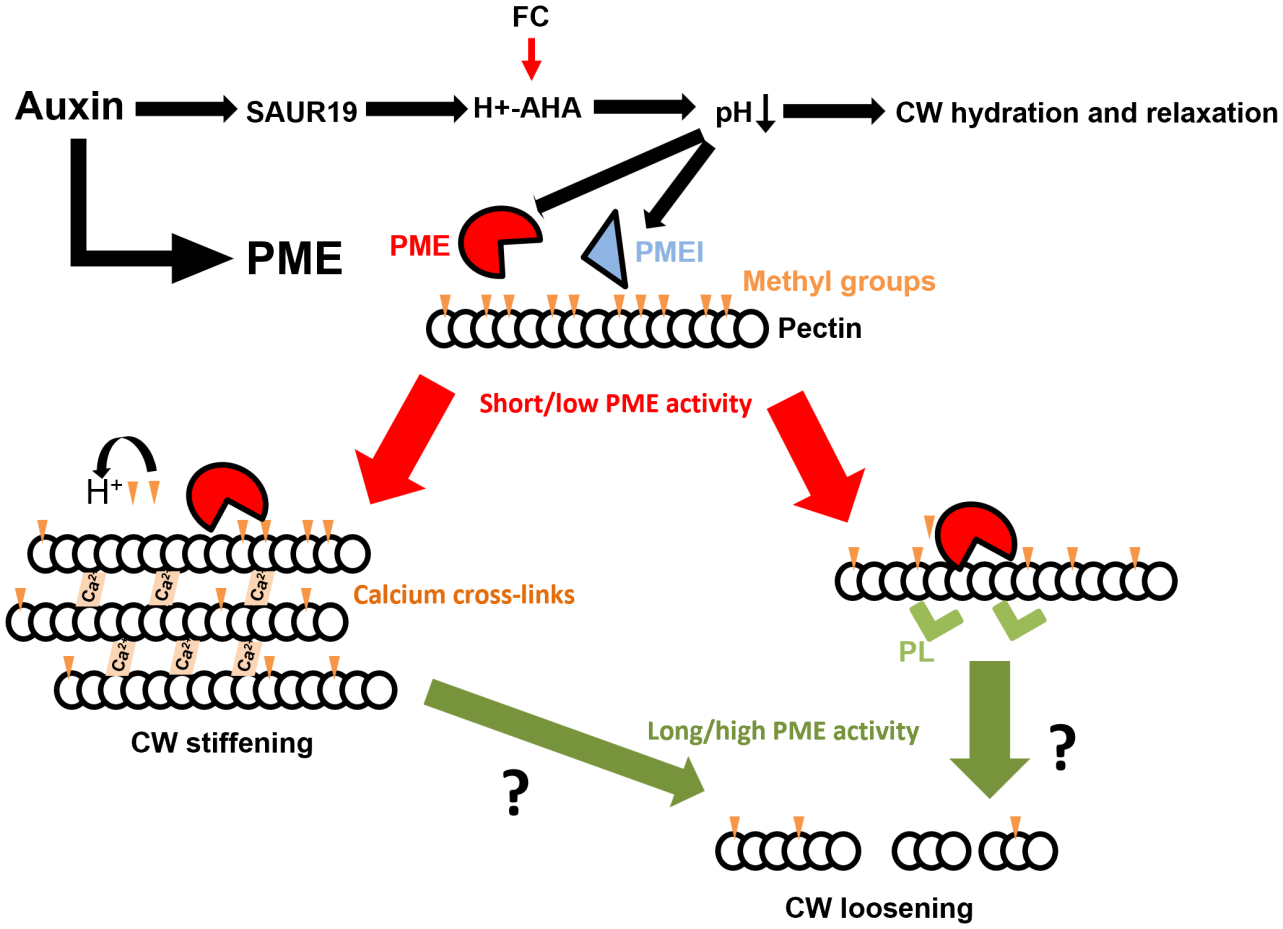
Model for the dual role of PME activity. Hormonal treatments converge on the acidification of apoplast, which trigger a decrease in CW stiffness. In parallel, short-term PME activation might generate a pectin de-methylesterification pattern that enhances calcium-mediated cross-linking and stiffening of CW, whereas long-lasting PME activity might induce pectin degradation and CW softening. Partially adapted from Hocq et al. (2017).

However, experimental observations showing that enhanced PME activity might lead to both reduced or increased CW stiffness raised a question about underlying mechanisms. This led to the formulation of a model proposing that the distinct outputs might be dependent on the pattern of pectin- de-methylation by PME (Hocq et al., 2017). Our experiments support the model in which PME long-term or high activity induces softening of CWs probably because loss of methyl groups might allow pectin lyases and other degradation enzymes to break long pectin polysaccharide chains, while short-term or moderate PME activity leads to CW stiffening as a result of pectin cross-linking by calcium (**Figure 5**). However, overexpression of enzymes like PMEs might lead to compensatory mechanisms that trigger the opposite effect (i.e., an overexpression of *PMEI* that led to a lower PME activity). Therefore, more research to demonstrate the degradation of pectin should be done, for example, staining and measuring pectin fibers before and after PME pulse, oligosaccharide quantifications, or studying the expression of PMEIs after PME induction. Future works will have to shed light on that point.

Notably, hypocotyl elongation assays are typically performed under calcium depletion, which might create artificial conditions under which pectin is not able to form new calcium cross-links due to the deficiency of calcium ions. Indeed our results show that adding calcium might interfere with auxin-triggered CW softening and elongation processes. Furthermore, it is proposed that acidification and CW softening is correlated with calcium import (Cho et al., 2012; Simon et al., 2011) as a result of cell expansion that might stretch the plasma membrane and open stretch-activated calcium channels.

Overall, we have demonstrated that acidification and growth processes mediated by several hormones converge on the CW softening process and that regulation of the PME activity tightly linked with extracellular calcium levels are essential factors determining the CW mechanical properties. Further studies characterizing the effects of PMEs and expansin proteins on the CW fiber interactions and re-arrangements might shed light on the complex network of CW remodeling in growth processes.

## Materials and methods

### Plant material

*Arabidopsis thaliana* plants were grown in a growth chamber at 21°C under white light (W), which was provided by blue and red LEDs (70–100 µmol m^-2^ s^-1^ of photosynthetically active radiation). The transgenic lines have been described elsewhere: *HS*::*axr3-1* (Knox et al., 2003), *35S::GFP-SAUR19* (Spartz et al., 2012), R2D2 (Liao et al., 2015), and ApopH (Fendrych et al., 2016). The *HS::axr3–1* plants were heat-shocked at 37°C for 40 min in aluminum-wrapped petri dishes; the experiments were started 1.5 h after the end of the heat shock.

All cloning and transformation procedures were conducted using Gateway™ (Invitrogen) technology, with the sequences of all vectors used available online (https://gateway.psb.ugent.be/), similarly as described before (Andrej et al., 2020). Inducible *PMEox* lines were generated by PCR amplification of cDNA from 3-day-old seedlings and insertion on pDONR221™ B1-B2 Gateway vector. The primers used for cloning can be found in **Supplementary Table S1**. The inserts were confirmed by PCR and sequencing. Afterward, the clones were recombined into pDEST™ R4-R3 pB-rPPT, together with the promoter from pUBQ10-XVE-P4P1 and the tag from pEN-R2-3xHA-L3 vectors. All transgenic plants were generated by the floral dip method in Columbia (Col-0) background, and transformants were selected on plates with respective antibiotic. Induction by β-Est was done by transferring the seedlings in AM plate with indicated concentrations of β-Est at indicated times.

### Growth conditions

Seeds were sterilized in 5% bleach for 10 min and rinsed with sterile water before plating on half-strength Murashige and Skoog (MS) medium (Duchefa) with 1% sucrose and 1% agar (pH 5.7). The seeds were stratified for 3 to 4 days at 4°C, exposed to light for 2–4 hours at 21°C, and cultivated in the growth chamber under appropriate light conditions at 21°C (wrapped in aluminum foil and disposed in a cardboard box for dark growth or into light in the same chamber).

### Elongation assays

Hypocotyl elongation assays were performed as described elsewhere (Fendrych et al., 2016) with minor modifications. Hypocotyl segments were obtained from etiolated seedlings ~72 h old (starting after transferring the plates into the growth room) by removing the hook and the lower half part of the hypocotyl (and root) using a razor blade in the darkness (a green LED light was used for illumination). The hypocotyl segments were placed on the surface of the depletion medium (DM; 10 mM KCl, 1 mM MES, pH 6,0 using KOH, 1.5% phytagel) and kept in the darkness for at least 20 min. The pH was adjusted using HCl or NaOH at 10 mM. Calcium and magnesium were added as CaCl_2_ or MgCl_2_ (Sigma-Aldrich) at indicated concentrations. Then, the segments were transferred to a new plate with depletion medium and the supplemented hormones or inhibitors as specified in the figure legends. The samples were placed on a flatbed scanner (Epson perfection V370) and imaged through the layer of phytagel, and a black filter paper was placed above the dishes to improve the contrast of the images. Samples were scanned immediately after transfer (used as initial length) and, unless under other specifications, after 3 h of incubation in the respective treatment. Final length was divided by initial length to obtain the relative elongation rate and normalize growth independently of absolute segment length.

### Root lengths

ImageJ software (http://rsb.info.nih.gov/) was used on digital images to measure the root length of 3-day-old dark-grown seedlings not treated or treated with 5 µM β-Est for 48 h (the 24-h-old seedlings were transferred to a new AM plate containing or not β-Est and incubated for 48 h in the darkness).

### Chemicals used

Auxin, 1-naphthaleneacetic acid (NAA), epibrassinolide (eBL), gibberellic acid (GA), fusicoccin (FC), β-estradiol (β-Est), and ethylene glycol-bis(β-aminoethyl ether)-N,N,N′,N′-tetraacetic acid (EGTA) were ordered from Sigma-Aldrich. NAA and eBL were used at 10 mM, and GA was dissolved to 100-mM stock concentration and FC at 1 mM in EtOH. β-Est was dissolved in water at 10 mM. EGTA was prepared in stock solution at 0.5 M in water.

### Confocal imaging

Confocal laser scanning micrographs were obtained with a Zeiss LSM800 with a 488-nm argon laser line for excitation of GFP fluorescence. Emissions were detected between 505 and 580 nm. Using a ×20 air objective, confocal scans were performed with the pinhole at 1 Airy unit. Localization was examined by confocal z-sectioning. Each image represents either a single focal plane or a projection of individual images taken as a z-series. Z-stacking was performed by collecting images through the cortex and epidermal layers. Full z-stack confocal images were 3D-projected using ImageJ software. At least five seedlings were analyzed per treatment. The images were processed and quantified in ImageJ. For ApopH, regions of interest (ROIs) were defined at the plasma membrane using a manual line of ImageJ tool, and for R2D2 individual nuclei were selected as ROIs using manual selection with a round shape.

### AFM measurements and apparent Young’s modulus calculations

The AFM data were collected and analyzed as described elsewhere with minor changes (Peaucelle et al., 2015; Andrej et al., 2020; Silvia et al., 2021). To examine extracellular matrix properties, we suppressed the turgor pressure by immersion of the seedlings in a hypertonic solution (10% mannitol) for at least 20 min before examination. Three-day-old seedlings grown in darkness (in normal AM plate or indicated treatment) or hypocotyl segments treated as described for elongation assays were placed in microscopy slides and immobilized using double-glued side tape. We focused on the periclinal CWs (parallel to the growth axis but perpendicular to the organ surface and perpendicular to the indentation direction) and its extracellular matrix. To ensure proper indentations, especially on the regions in the bottom of the dome shape between two adjacent cells, we used cantilevers with a long pyramidal tip (14–16 μm of pyramidal height, AppNano ACST-10), with a spring constant of 7.8 N/m. All cantilevers were calibrated prior to the biomechanical experiments. The stiffness of the cantilever was calibrated by thermal noise measurement, and the sensitivity was calibrated by force–distance curve measurement with a microscopy glass slide (setpoint value, 1.5 V). The instrument used was a JPK Nano-Wizard 4.0, and indentations were kept to <10% of the cell height (typically indentations of 100–200-nm depth and 500-nN force). Typically, three different scan-maps per sample were taken over an intermediate region of the hypocotyl, using a square area of 25 × 25 μm, with 16 × 16 force-indentation measurements. The lateral deflection of the cantilever was monitored, and in case of any abnormal increase, the entire data set was not used for the analysis. The apparent Young’s modulus (*E_a_*) for each force-indentation experiment was calculated using the approach curve (to avoid any adhesion interference) with the JPK Data Processing software (JPK Instruments AG, Germany) based on Hertz model adjusted to pyramidal tip geometry. To calculate the average *E_a_* for each periclinal wall, the *E_a_* was measured over the total length of the extracellular region using masks with Gwyddion 2.45 software (at least 20 points were taken into account). The pixels corresponding to the extracellular matrix were chosen based on topography and Young’s modulus maps. For topographical reconstructions, the height of each point was determined by the point-of-contact from the force-indentation curve. A standard *t*-test was applied to test for differences between genotypes/treatments. The total number of scans performed and analyzed (i.e., independent cell walls measured) is indicated in the figures (indicated as *n*).

### Expression analysis by RT-qPCR

Around 60 seedlings (72-h-old, dark-grown of indicated lines, transferred after 48 h to AM plates containing or not 2 µM β-Est) were harvested and frozen in liquid nitrogen. Tissue was ground using a ball mill (model MM400; Retsch) with 4-mm diameter balls in a 2-mL Eppendorf (Hamburg, Germany). Total RNA was isolated using Monarch kit (New England Biolabs) according to the manufacturer’s protocol (DNase treatment was included during the extraction protocol). cDNA was prepared from 1 µg of total RNA with the iScript cDNA Synthesis Kit (Biorad), diluted 10 times, and 1 µL was used in a 5-µL PCR reaction on a LightCycler 480 (Roche Diagnostics) with Luna Master Mix (New England Biolabs) according to the manufacturer’s instructions. As control, non-RT-treated samples were included to test the purity of the cDNA. All experiments were done with three technical replicates and three biological samples. The PP2A gene (At1g69960) was used as a control for normalizations. The primer sequences can be found in **Supplementary Table S1**.

### Western blot analysis

Around 60 seedlings (72-h-old, dark-grown of indicated lines, transferred after 48 h to AM plates containing or not 2 µM β-Est) were harvested and frozen in liquid nitrogen. Frozen tissue was ground with stainless steel 4-mm-diameter balls in a 2-mL Eppendorf (Hamburg, Germany) tube using a ball mill (model MM400; Retsch). Extraction buffer [100 mM Tris–HCl (pH 7.5), 25% (w/w, 0.81 M) sucrose, 5% (v/v) glycerol, 10 mM ethylenediaminetetraacetic acid (EDTA, pH 8.0), 10 mM ethyleneglycoltetraacetic acid (EGTA pH 8.0), 5 mM KCl, and 1 mM 1,4-dithiothreitol (DTT); Abas and Luschnig, 2010] was added to the frozen tissue. After the Bradford quantification, 20 µg of protein was diluted in 20 µL of buffer and prepared for electrophoresis by adding 5 µL of loading buffer (5× SDS) and incubating at 45°C for 5 min. A run was performed in a commercial 10% Mini-PROTEAN^®^ TGX™ Precast Protein Gels (Bio-Rad) at 35 mA. Transference was done with a semidry system with a Trans-blot turbo transfer pack PVDF (Bio-Rad). The blot was washed in TBST buffer with 5% milk powder and 0.05% Tween20 and blocked overnight in the same buffer at 4°C. Hybridization was done with anti-HA-HRP antibody (monoclonal antibody from Sigma, dilution 1:7,000) in TBST for 2 h at room temperature. The blot was washed three times in TBST and once in water, with previous visualization by SuperSignal West Femto Maximum Sensitivity Substrate kit (Thermo Scientific) and exposure to Amersham Imager 600 (GE Healthcare).

### Immunostaining

Immunostaining was performed as described elsewhere (Taras et al., 2015). Briefly, 72-h dark-grown seedlings of indicated lines (transferred after 48 h to AM plates containing or not 2 µM β-estradiol) were fixed using methanol for 20 min at 37°C, followed by 3 min at 60°C incubation. Water was added slowly until the methanol concentration reached 20%, and the seedlings were transferred to the water solution. CW was digested using 0.2% driselase and 0.15% macerozyme in 2 mM MES, pH 5.0 (37°C, 40 min), and membrane permeabilization using 3% NP‐40 and 10% DMSO in 1× MTSB (37°C, 20 min). LM19 (1:50, Plantprobes) was used as a primary antibody (4°C, o/v). Alexa Fluor 488 goat anti‐rat (Thermo Fischer Scientific) was used as secondary antibody (1:500) (room temperature, 60 min). Finally, the samples were mounted in VECTASHIELD^®^ Antifade Mounting Medium. Images were obtained using an LSM800 microscope as explained above.

### Accession numbers

The sequence data from this article can be found in the Arabidopsis Genome Initiative or GenBank/EMBL databases under the following accession numbers: At1g53840 (*PME1*) and At5g47500 (*PME5*).

## Data Availability

The datasets presented in this study can be found in online repositories. The names of the repository/repositories and accession number(s) can be found below: https://www.biorxiv.org/content/10.1101/2022.06.14.495617v1.

## References

[B1] Abas L. Luschnig C.. Maximum yields of microsomal-type membranes from small amounts of plant material without requiring ultracentrifugation. Anal Biochem. (2010) 401(2), 217–27. doi: 10.1016/j.ab.2010.02.030 20193653 PMC3685806

[B2] Andrej H. Candela C. Nicola C. Krisztina Ö. Jerome D. Ladislav D. (2020). SYNERGISTIC ON AUXIN AND CYTOKININ 1 positively regulates growth and attenuates soil pathogen resistance. Nat. Commun. 11. doi: 10.1038/s41467-020-15895-5 PMC7195429 32358503

[B3] Arsuffi G. Braybrook S. A. (2018). Acid growth: an ongoing trip. J. Exp. Bot. 69, 137–146. doi: 10.1093/jxb/erx390 29211894

[B4] Ballio A. EB C. P D. L. BF E. M M. A. T. (1964). Fusicoccin: a New Wilting Toxin produced by Fusicoccum amygdali Del. Nat. 223 397, 4942. doi: 10.1038/203297a0

[B5] Bates G. W. Cleland R. E. (1979). Protein synthesis and auxin-induced growth: Inhibitor studies. Planta 145, 437–442. doi: 10.1007/BF00380097 24317859

[B6] Baunsgaard L. Fuglsang A. T. Jahn T. Korthout H. A. A. J. De Boer A. H. Palmgren M. G. (1998). The 14-3–3 proteins associate with the plant plasma membrane H+-atpase to generate a fusicoccin binding complex and a fusicoccin responsive system. Plant J. 13, 661–671. doi: 10.1046/j.1365-313X.1998.00083.x 9681008

[B7] Bou Daher F. Chen Y. Bozorg B. Clough J. Jönsson H. Braybrook S. A. (2018). Anisotropic growth is achieved through the additive mechanical effect of material anisotropy and elastic asymmetry. Elife 7, e38161. doi: 10.7554/eLife.38161 30226465 PMC6143341

[B8] Braidwood L. Breuer C. Sugimoto K. (2014). My body is a cage: Mechanisms and modulation of plant cell growth. New Phytol. 201, 388–402. doi: 10.1111/nph.2013.201.issue-2 24033322

[B9] Caesar K. Elgass K. Chen Z. Huppenberger P. Witthöft J. Schleifenbaum F.. (2011). A fast brassinolide-regulated response pathway in the plasma membrane of Arabidopsis thaliana. Plant J. 66, 528–540. doi: 10.1111/j.1365-313X.2011.04510.x 21255166

[B10] Cho D. Villiers F. Kroniewicz L. Lee S. Seo Y. J. Hirschi K. D.. (2012). Vacuolar CAX1 and CAX3 influence auxin transport in guard cells via regulation of apoplastic pH. Plant Physiol. 160, 1293–1302. doi: 10.1104/pp.112.201442 22932758 PMC3490596

[B11] Cleland R. E. Buckley G. Nowbar S. Lew N. M. Stinemetz C. Evans M. L.. (1991). The pH profile for acid-induced elongation of coleoptile and epicotyl sections is consistent with the acid-growth theory. Planta 186, 70–74. doi: 10.1007/BF00201499 11538124

[B12] Cosgrove D. J. (1993). Wall extensibility: its nature, measurement and relationship to plant cell growth. New Phytol. 124, 1–23. doi: 10.1111/j.1469-8137.1993.tb03795.x 11537718

[B13] Cosgrove D. J. (2018). Nanoscale structure, mechanics and growth of epidermal cell walls. Curr. Opin. Plant Biol. 46, 77–86. doi: 10.1016/j.pbi.2018.07.016 30142487

[B14] Cosgrove D. J. (2022). Building an extensible cell wall. Plant Physiol. 189, 1246–1277. doi: 10.1093/plphys/kiac184 35460252 PMC9237729

[B15] Elsayad K. Werner S. Gallemí M. Kong J. Guajardo E. R. S. Zhang L.. (2016). Mapping the subcellular mechanical properties of live cells in tissues with fluorescence emission – Brillouin imaging. Sci. Signal. 9, 1–13. doi: 10.1126/scisignal.aaf6326 27382028

[B16] Fendrych M. Leung J. Friml J. (2016). TIR1/AFB-Aux/IAA auxin perception mediates rapid cell wall acidification and growth of Arabidopsis hypocotyls. Elife 10, 53–59. doi: 10.7554/eLife.19048.019 PMC5045290 27627746

[B17] Feng W. Kita D. Peaucelle A. Wu H. (2018). The FERONIA Receptor Kinase Maintains Cell-Wall Integrity during Salt Stress through Ca 2 + Signaling Article The FERONIA Receptor Kinase Maintains Cell-Wall Integrity during Salt Stress through Ca 2 + Signaling. Curr Biol. 28, 666–675. doi: 10.1016/j.cub.2018.01.023 29456142 PMC5894116

[B18] Gjetting S. K. Ytting C. K. Schulz A. Fuglsang A. T. (2012). Live imaging of intra- and extracellular pH in plants using pHusion , a novel genetically encoded biosensor. J Exp Bot. 63(8), 3207–18. doi: 10.1093/jxb/ers040 22407646 PMC3350929

[B19] Haas K. T. Wightman R. Meyerowitz E. M. Peaucelle A. (2020). Pectin homogalacturonan nanofilament expansion drives morphogenesis in plant epidermal cells. Sci. (80-.) 367, 1003–1007. doi: 10.1126/science.aaz5103 PMC7932746 32108107

[B20] Haas K. T. Wightman R. Peaucelle A. Herman H. (2021). The role of pectin phase separation in plant cell wall assembly and growth. Cell Surf 7, 2468–2330. doi: 10.1016/j.tcsw.2021.100054 PMC8185244 34141960

[B21] Hocq L. Pelloux J. Lefebvre V. (2017). Connecting homogalacturonan-type pectin remodeling to acid growth. Trends Plant Sci. 22, 20–29. doi: 10.1016/j.tplants.2016.10.009 27884541

[B22] Irabonosi O. Iain J. P. Peter L.J. Fabienne B. Nathan R. Chieko K.I. (2025). Understanding pectin cross-linking in plant cell walls. Commun. Biol. 8, 72. doi: 10.1038/s42003-025-07495-0 39825091 PMC11748717

[B23] Jonsson K. Hamant O. Bhalerao R. P. (2022). Plant cell walls as mechanical signaling hubs for morphogenesis. Curr. Biol. 32, R334–R340. doi: 10.1016/j.cub.2022.02.036 35413265

[B24] Jonsson K. Lathe R. S. Kierzkowski D. Routier-Kierzkowska A. L. Hamant O. Bhalerao R. P. (2021). Mechanochemical feedback mediates tissue bending required for seedling emergence. Curr. Biol. 31, 1154–1164.e3. doi: 10.1016/j.cub.2020.12.016 33417884

[B25] Knox K. Grierson C. S. Leyser O. (2003). AXR3 and SHY2 interact to regulate root hair development. Development 130, 5769–5777. doi: 10.1242/dev.00659 14534134

[B26] Kutschera U. Schopfer P. (1985). Evidence for the acid-growth theory of fusicoccin action. Planta 163, 494–499. doi: 10.1007/BF00392706 24249448

[B27] Liao C. Y. Smet W. Brunoud G. Yoshida S. Vernoux T. Weijers D. (2015). Reporters for sensitive and quantitative measurement of auxin response. Nat. Methods 12, 207–210. doi: 10.1038/nmeth.3279 25643149 PMC4344836

[B28] Lin W. Zhou X. Tang W. Takahashi K. Pan X. Dai J.. (2021). TMK-based cell-surface auxin signalling activates cell-wall acidification. Nature. 599(7884), 278–282. doi: 10.1038/s41586-021-03976-4 34707287 PMC8549421

[B29] Lionetti V. Francocci F. Ferrari S. Volpi C. Bellincampi D. Galletti R.. (2010). Engineering the cell wall by reducing de-methyl-esterified homogalacturonan improves saccharification of plant tissues for bioconversion. Proc. Natl. Acad. Sci. U. S. A. 107, 616–621. doi: 10.1073/pnas.0907549107 20080727 PMC2818903

[B30] Lionetti V. Raiola A. Camardella L. Giovane A. Obel N. Pauly M.. (2007). Overexpression of pectin methylesterase inhibitors in Arabidopsis restricts fungal infection by Botrytis cinerea. Plant Physiol. 143, 1871–1880. doi: 10.1104/pp.106.090803 17277091 PMC1851811

[B31] Liu N. Sun Y. Pei Y. Zhang X. Wang P. Li X.. (2018). A pectin methylesterase inhibitor enhances resistance to verticillium wilt. Plant Physiol. 176, 2202–2220. doi: 10.1104/pp.17.01399 29363564 PMC5841709

[B32] Ludivine H. Fabien S. Valérie L. Arnaud L. Jean-Marc D. Jean-Claude M. (2017). Combined experimental and computational approaches reveal distinct pH dependence of pectin Methylesterase Inhibitors. Plant Physiol. 173, 1075–1093. doi: 10.1104/pp.16.01790 28034952 PMC5291010

[B33] Majda M. Robert S. (2018). The role of auxin in cell wall expansion. Int. J. Mol. Sci. 19:951. doi: 10.3390/ijms19040951 29565829 PMC5979272

[B34] Minami A. Takahashi K. Inoue S. Tada Y. Kinoshita T. (2019). Brassinosteroid Induces Phosphorylation of the Plasma Membrane H+-ATPase during Hypocotyl Elongation in Arabidopsis thaliana. Plant Cell Physiol. 60, 935–944. doi: 10.1093/pcp/pcz005 30649552

[B35] Mockaitis K. Estelle M. (2008). Auxin receptors and plant development: A new signaling paradigm. Annu Rev Cell Dev Biol. 24, 55–80. doi: 10.1146/annurev.cellbio.23.090506.123214 18631113

[B36] Nemhauser J. L. Hong F. Chory J. (2006). Different plant hormones regulate similar processes through largely nonoverlapping transcriptional responses. Cell 126, 467–475. doi: 10.1016/j.cell.2006.05.050 16901781

[B37] Oh E. Zhu J. Y. Bai M. Y. Arenhart R. A. Sun Y. Wang Z. Y. (2014). Cell elongation is regulated through a central circuit of interacting transcription factors in the Arabidopsis hypocotyl. Elife 2014, 1–19. doi: 10.7554/eLife.03031.025 PMC4075450 24867218

[B38] Olivier A. Ibrahim C. Benoit L. Yuchen L. (2022). Revisiting the relationship between turgor pressure and plant cell growth. New Phytol. 238, 62–69. doi: 10.1111/nph.v238.1 36527246

[B39] Peaucelle A. Braybrook S. A. Le Guillou L. Bron E. Kuhlemeier C. Höfte H. (2011). Pectin-induced changes in cell wall mechanics underlie organ initiation in Arabidopsis. Curr. Biol. 21, 1720–1726. doi: 10.1016/j.cub.2011.08.057 21982593

[B40] Peaucelle A. Louvet R. Johansen J. N. Höfte H. Laufs P. Pelloux J.. (2008). Arabidopsis phyllotaxis is controlled by the methyl-esterification status of cell-wall pectins. Curr. Biol. 18, 1943–1948. doi: 10.1016/j.cub.2008.10.065 19097903

[B41] Peaucelle A. Wightman R. Höfte H. (2015). The control of growth symmetry breaking in the arabidopsis hypocotyl. Curr. Biol. 25(13), 1746–1752. doi: 10.1016/j.cub.2015.05.022 26073136

[B42] Perrot-rechenmann C. (2010). Cellular responses to auxin: division versus expansion. Cold Spring Harb Perspect Biol. 2 (5), a001446. doi: 10.1101/cshperspect.a001446 20452959 PMC2857164

[B43] Rayle D. L. Cleland R. E. (1980). Evidence that auxin-induced growth of soybean hypocotyls involves proton excretion. Plant Physiol. 66, 433–437. doi: 10.1104/pp.66.3.433 16661450 PMC440648

[B44] Rayle D. L. Cleland R. E. (1992). The Acid Growth Theory of auxin-induced cell elongation is alive and well. Plant Physiol. 99, 1271–1274. doi: 10.1104/pp.99.4.1271 11537886 PMC1080619

[B45] Röckel N. Wolf S. Kost B. Rausch T. Greiner S. (2008). Elaborate spatial patterning of cell-wall PME and PMEI at the pollen tube tip involves PMEI endocytosis, and reflects the distribution of esterified and de-esterified pectins. Plant J. 53, 133–143. doi: 10.1111/j.1365-313X.2007.03325 17971035

[B46] Rose R. J. (1974). Differntial effect of cycloheximide on the short term gibberellin and auxin growth kinetics of gamma-coleoptiles. Plant Sci. Lett. 2, 233–237. doi: 10.1016/0304-4211(74)90121-7

[B47] Saffer A. M. (2018). Expanding roles for pectins in plant development. J. Integr. Plant Biol. 60, 910–923. doi: 10.1111/jipb.v60.10 29727062

[B48] Schenck D. Christian M. Jones A. Luthen H. (2010). Rapid auxin-induced cell expansion and gene expression: A four-decade-old question revisited. Plant Physiol. 152, 1183–1185. doi: 10.1104/pp.109.149591 20071604 PMC2832252

[B49] Shin Y. Chane A. Jung M. Lee Y. (2021). Recent advances in understanding the roles of pectin as an active participant in plant signaling networks. Plants (Basel Switzerland) 10:1712. doi: 10.3390/plants10081712 34451757 PMC8399534

[B50] Silvia M. V. Xiaoyuan G. Marçal G. Bibek A. Peter V. Elke B.. (2021). Xyloglucan remodeling defines auxin-dependent differential tissue expansion in plants. Int. J. Mol. Sci. 22. doi: 10.3390/ijms22179222 PMC8430841 34502129

[B51] Simon J. C. Matthew G. Asmini A. Andreas W. S. Ute B. (2011). Cell-specific vacuolar calcium storage mediated by CAX1 regulates apoplastic calcium concentration, gas exchange, and plant productivity in Arabidopsis. Plant Cell 23, 240–257. doi: 10.1105/tpc.109.072769 21258004 PMC3051233

[B52] Simonini S. Bencivenga S. Trick M. Østergaard L. (2017). Auxin-induced modulation of ETTIN activity orchestrates gene expression in arabidopsis. Plant Cell 29, 1864–1882. doi: 10.1105/tpc.17.00389 28804059 PMC5590509

[B53] Spartz A. K. Lee S. H. Wenger J. P. Gonzalez N. Itoh H. Inzé D.. (2012). The SAUR19 subfamily of SMALL AUXIN UP RNA genes promote cell expansion. Plant J. 70, 978–990. doi: 10.1111/j.1365-313X.2012.04946.x 22348445 PMC3481998

[B54] Steffen V. Bert D. R. Gerrit T. S. B. Karin L. Ive D. S. Gert V. I.. (2005). Cell cycle progression in the pericycle is not sufficient for SOLITARY ROOT/IAA14-mediated lateral root initiation in Arabidopsis thaliana. Plant Cell 17, 3035–3050. doi: 10.1105/tpc.105.035493 16243906 PMC1276028

[B55] Taras P. Olaf T. Katja R. Maura B. Roland N. Benedetto R.. (2015). Protocol: an improved and universal procedure for whole-mount immunolocalization in plants. Plant Methods 11, 50. doi: 10.1186/s13007-015-0094-2 26516341 PMC4625903

[B56] Tode K. Lüthen H. (2001). Fusicoccin- and IAA-induced elongation growth share the same pattern of K+ dependence. J. Exp. Bot. 52, 251–255. 11283169

[B57] Velasquez S. M. Barbez E. Kleine-Vehn J. Estevez J. (2016). Auxin and cellular elongation. Plant Physiol. 22(17), 01863.2015. doi: 10.1104/pp.15.01863 PMC4775141 26787325

[B58] Wolf S. Hématy K. Höfte H. (2012). Growth control and cell wall signaling in plants. Annu. Rev. Plant Biol. 63, 381–407. doi: 10.1146/annurev-arplant-042811-105449 22224451

[B59] Zhang T. Tang H. Vavylonis D. Cosgrove D. J. (2019). Disentangling loosening from softening: insights into primary cell wall structure. Plant J. 100, 1101–1117. doi: 10.1111/tpj.v100.6 31469935

