# Supplementary material for: Dual role of pectin methyl esterase activity in the regulation of plant cell wall biophysical properties
Source: Front Plant Sci. 2025 Jul 4;16:1612366. doi: 10.3389/fpls.2025.1612366 (PMC12271113; doi:10.3389/fpls.2025.1612366)
Supplement: Supplementary file 1 [file DataSheet1.pdf]

Supplementary Figure 1

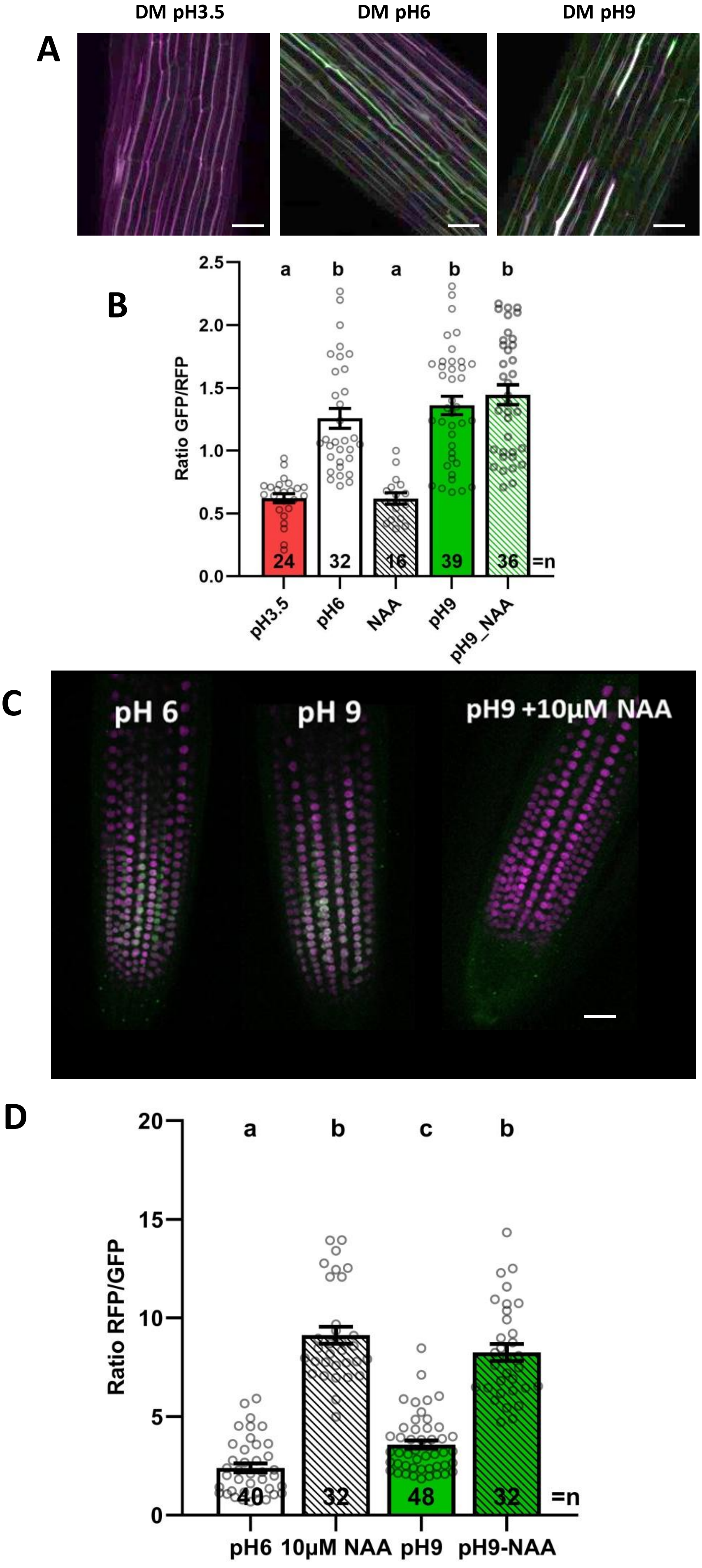

Supplementary Figure 1. Effect of pH buffered media on apoplastic region and auxin signaling. A and B. Hypocotyl segments of 3 days-old seedlings expressing ApopH marker line (GFP/RFP) incubated in buffered depletion medium (DM) with pH as indicated for 2 h. Maximum intensity Z-stack projection images (A) and apoplast pH quantified as GFP/RFP intensity ratio. Scale bar = 50 µm (B). C and D. 3 days-old roots expressing the *R2D2* auxin signaling reporter 2 h after indicated treatment. Maximum intensity Z-stack projection images of root meristematic zone (C) and quantification of auxin response as RFP/GFP signal ratio. Scale bar = 25 µm (D). Bars represent average ±*SE*, dots represents individual data points. N indicates the total number of cells quantified. Tukey-Kramer test was performed and significant differences are shown as the letters above each bar.

Supplementary Figure 2

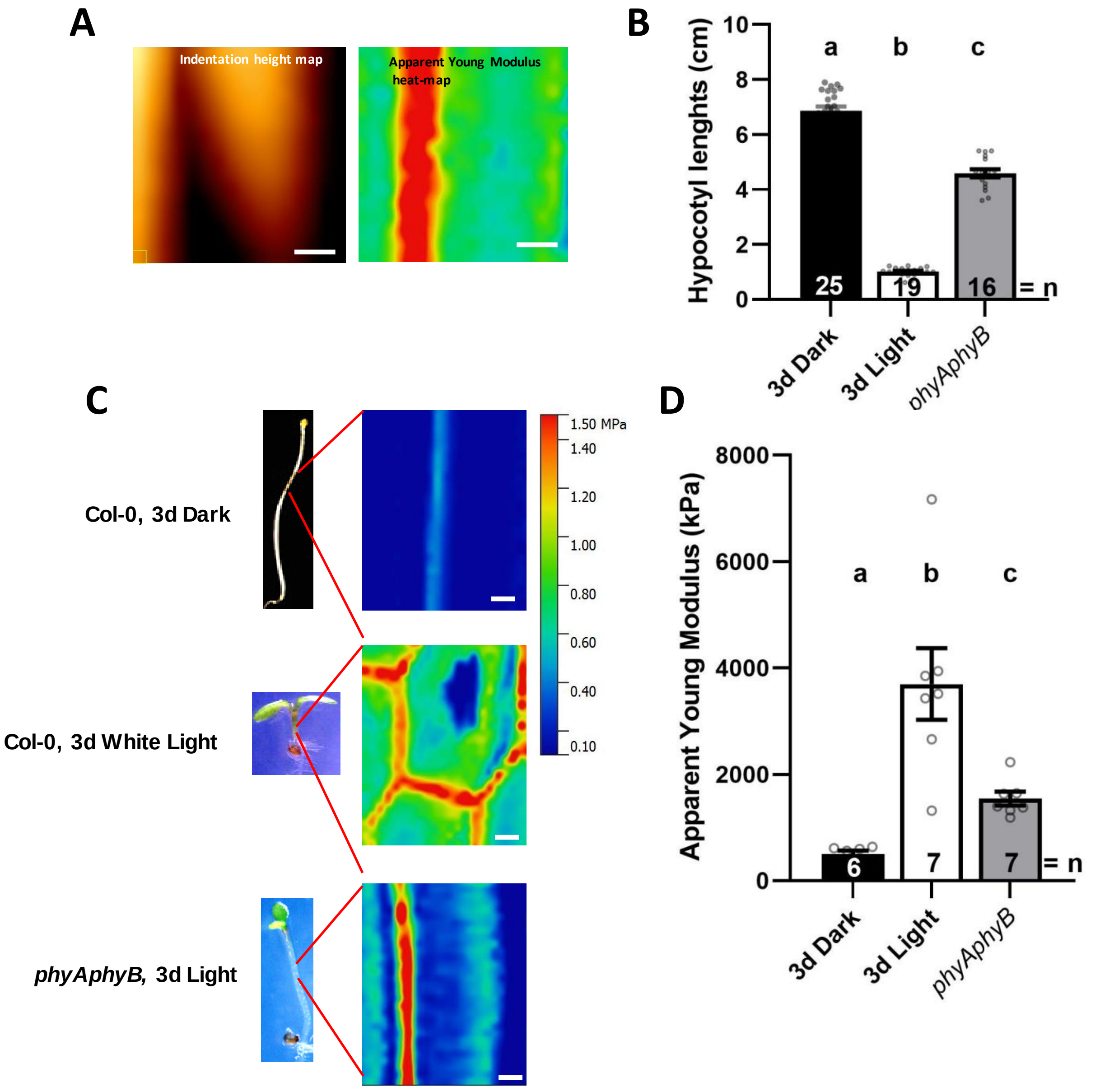

Supplementary Figure 2. Atomic force microscopy (AFM) analyses of cell wall stiffness in hypocotyl epidermal cells. A. Representative heat-maps of Indentation Height (left) and Apparent Young Modulus (E_a, right) of periclinal epidermal CWs. Scale bar = 5µm. B. Hypocotyl lengths of 3 days-old wild-type (Col-0) and *phyAphyB* seedlings grown in light or darkness. C. Representative AFM scans with Apparent Young Modulus (E_a) heat-map color coded of periclinal epidermal CWs in hypocotyls of Col-0 and *phyA,phyB*. Scale bar = 5µm. D. Quantifications of Average E_a for selected CW regions of corresponding heat-maps. Bars represent average ±*SE*, dots represents individual data points. N indicates the total number of hypocotyls measured (A) or individual periclinal cell wall scans performed and quantified (C). Tukey -Kramer test was performed and significant differences are shown as the letters above each bar.

Supplementary Figure 3

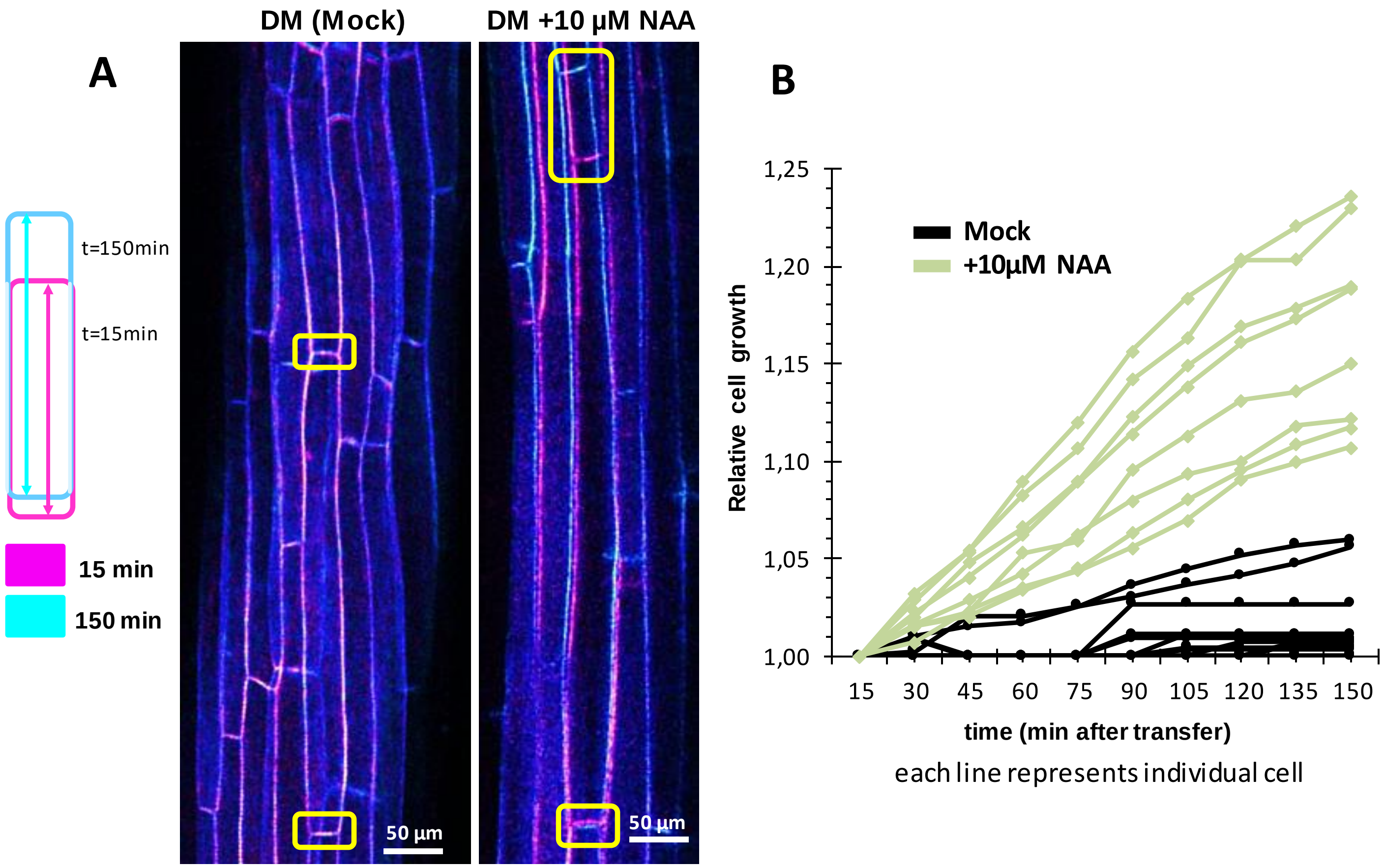

Supplementary Figure 3. Auxin promotes hypocotyl epidermal cell elongation.
A. A color code representation of hypocotyl epidermal cell elongation. Cell length at 15 min and 150 min, magenta and cyan, respectively, after incubation in the depletion medium (DM) and DM supplemented with auxin (1-naphthaleneacetic acid, NAA). Images are maximum intensity Z-stack projection of 3 day-old hypocotyl segments from seedlings expressing *35S::PIP2-GFP* plasma membrane reporter. Yellow squares remark areas were cell elongation can be observed. Scale bar = 50 µm. B. Time course of epidermal cell length increase. Epidermal cells in the middle part of hypocotyl segments were monitored and measured in indicated time intervals (cells from 3 different hypocotyls are shown).

Supplementary Figure 4

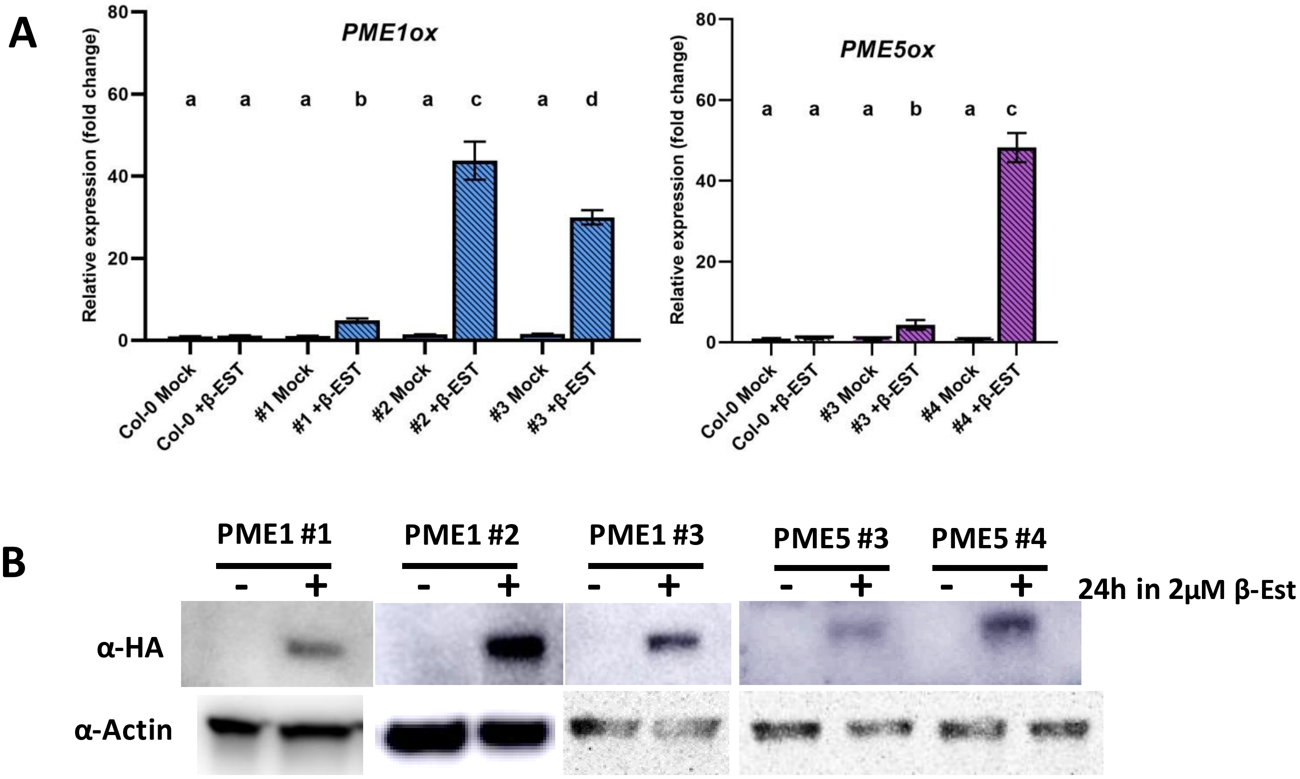

Supplementary Figure 4. Expression analyses of *PMEoxs* lines. A. Relative expression levels of *PME1ox* and *PME5ox* analysed by RT-qPCR in several independent homozygous transgenic lines. Expression of *PME*1 and *PME5* induced by treatment with 2 μM β-Est for 24 h. Expression monitored in whole 3 days-old dark grown seedlings. Tukey-Kramer test was performed and significant differences are shown as the letters above each bar. B. PME1-HA and PMA5-HA protein detected by Western-Blot analysis in several independent *PMEox*s transgenic lines induced by same treatments as mentioned before. α-actin monitored as a protein loading control.

Supplementary Figure 5

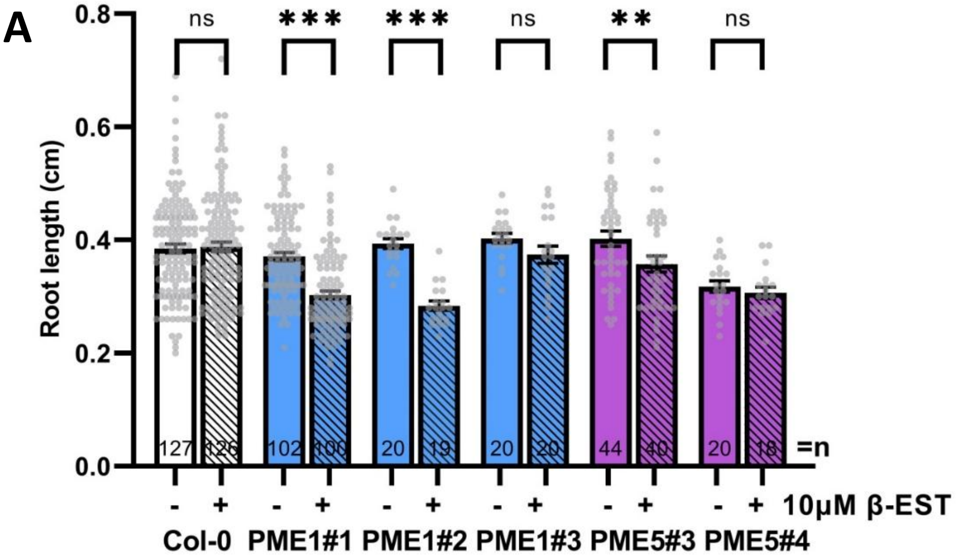

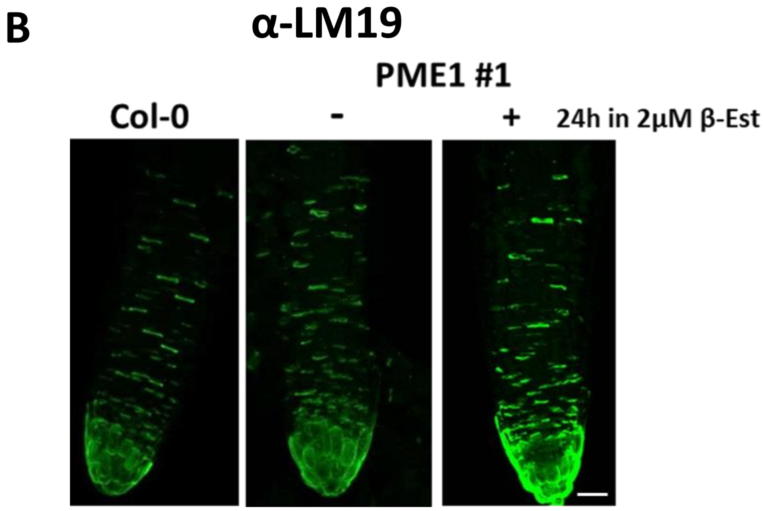

Supplementary Figure 5. Phenotype analyses of *PMEoxs* lines. A. Root lengths for several independent *PMEox*s transgenic lines. 3 days-old dark grown seedlings non- or treated with 10 μM β-Est for 48 h. Bars represent average ±*SE*, dots represents individual data points. N indicates the total number of roots quantified. Direct pair significant differences are indicated as **P < 0.01 and ***P < 0.001 (t-test). B. Maximum intensity Z-stack projection images of root tips immunoassayed with LM19 antibody for wild-type line and one PME1ox with and without expression induction in β.-Estradiol. Scale bar = 25 μm.

Supplementary Table S1. List of primers used

Name	Sequence	Gene\Seq	Purpuse
**MGR108_PP2A F**	TAACGTGGCCAAAATGATGC	AT1G69960	qPCR HK gene
**MGR109_PP2A R**	GTTCTCCACAACCGCTTGGT	AT1G69960	qPCR HK gene
**MGR112_PME1F**	ACGGTGATTGGAGCAGTGG	AT1G53840	qPCR PME1
**MGR113_PME1R**	CTGCGTTACATCTGAACCCG	AT1G53840	qPCR PME1
**MGR114_PME5F**	TCACCGTCTCACTTAACGGC	AT5G47500	qPCR PME5
**MGR115_PME5R**	TAGCGGTCACATCCCTACCA	AT5G47500	qPCR PME5
**MGR90_PME1Fw**	GGGGACAAGTTTGTACAAAAAAGCAGGCTCC_ATGGATTCAGTGAACTCCTTCAAAGG	AT1G53840	Cloning attB1 PME1
**MGR91b_PME1Rv**	GGGGACCACTTTGTACAAGAAAGCTGGGTGTA_AGATAGCTGATTGATCACTCCTGTTGC	AT1G53840	Cloning attB2 PME1
**MGR92_PME5Fw**	GGGGACAAGTTTGTACAAAAAAGCAGGCTCC_ATGGCGCAACTTACTAATTCCCTC	AT5G47500	Cloning attB1 PME5
**MGR93b_PME5Rv**	GGGGACCACTTTGTACAAGAAAGCTGGGTGTA_AGCATCTCGAGGAGCGATCC	AT5G47500	Cloning attB2 PME5
**MGR111_HARv**	CCGCATAGTCAGGAACATCG	HA tag	Genotypings
